# Supplementary material for: The potential deployment of a pan-tuberculosis drug regimen in India: A modelling analysis
Source: PLoS One. 2020 Mar 27;15(3):e0230808. doi: 10.1371/journal.pone.0230808 (PMC7100958; doi:10.1371/journal.pone.0230808)
Supplement: S1 Appendix — (DOCX) [file pone.0230808.s001.docx]

**The potential deployment of a pan-tuberculosis drug regimen in India: a modelling analysis**

N.Arinaminpathy, G.B.Gomez, K.S.Sachdeva, R.Rao, M.Parmar, S.A.Nair, K.Rade, S.Kumta, D.Hermann, C.Hanson, D.P.Chin, P.Dewan

Online supporting material

**Governing equations**

In the following governing equations, subscripts $i,j,k$ denote respectively: the setting (urban or rural); drug susceptibility status (drug-susceptible or drug resistant); and provider type (public or private). A list of compartment definitions ($U, L$, etc) is given in table S1. Model parameters are described in the text accompanying the equation where they first appear; they are also listed in table S2. Dots over characters represent derivatives with respect to time.

*Acquisition of infection*

$$\dot{U_{i}}=b-\left( \sum_{j} \lambda_{ij}-\mu\right)U_{i}$$

where $b$ is the rate of births in the population and $\mu$ is the ‘background’ hazard of mortality, unrelated to TB disease. Here, $\lambda_{ij}$ is the force-of-infection experienced by a susceptible individual from setting *i,* arising from strain *j.*

*Latent infection*

$$\dot{L}_{ij}=\left( 1-p_{fast} \right)\lambda_{ij}\left[ U_{i}+\omega\left( L_{ij}+R_{ij}^{\left( lo \right)}+R_{ij}^{\left( hi \right)}+S_{ij}^{\left( lo \right)}+S_{ij}^{\left( hi \right)} \right) \right]-(r+\mu)L_{ij}$$

where $p_{fast}$ is the proportion of infections being ‘fast’ progressors to active disease; $\omega$ is the protection to reinfection afforded by previous infection; $r$ is the per-capita rate of breakdown to active disease from latent infection.

*Active disease, pre-careseeking*

$$\dot{I_{ij}}=rL_{ij}+p_{fast}\lambda_{ij}\left[ U_{i}+\omega\left( L_{ij}+R_{ij}^{\left( lo \right)}+R_{ij}^{\left( hi \right)}+S_{ij}^{\left( lo \right)}+S_{ij}^{\left( hi \right)} \right) \right]+\sum_{reg} \left[ \gamma^{\left( lo,reg \right)}R_{ij}^{\left( reg \right)}+\gamma^{\left( hi,reg \right)}Q_{ij}^{\left( reg \right)} \right]+\gamma^{\left( stabilised \right)}C_{ij}-\left( d_{i}+\mu_{TB}+\sigma\right)I_{ij},$$

where the terms in $\gamma$ represent endogenous relapse from individuals with TB history (terms explained below); *d* is the mean per-capita rate of first presentation for care (to either the public or private sectors); $\mu_{TB}, \sigma$ are respectively the mean per-capita hazards of mortality and spontaneous cure, for untreated TB.

*Presenting for diagnosis in either public or private sectors*

$$\dot{D_{ijk}}=dw_{k}I_{ij}+ew_{k}E_{ij}-(r_{Dx}+\sigma)D_{ijk},$$

where $w_{k}$ is the proportion of symptomatics visiting provider type $k$; *e* is the per-capita rate at which TB cases who have dropped out of the care cascade re-engage in care; and $r_{Dx}$ is the mean per-capita rate at which a provider offers a diagnosis.

*Treatment course, different regimens (denoted by ‘reg’):*

$$\dot{T}_{ijk}^{\left( reg \right)}=r_{Dx}{\rho_{ijk}^{\left( reg \right)}D}_{ijk}+\delta_{j}^{(reg)}mT_{\left\{ i,DS,k \right\}}^{\left( FL \right)}- \left( \tau^{\left( reg \right)}+v_{k}^{\left( reg \right)}+\mu\right)T_{ijk}^{\left( reg \right)}$$

Here, *reg* can be ‘FL’ (first-line therapy); ‘SL’ (second-line therapy); or ‘ptr’ (pan-TB regimen). $\rho_{ijk}^{(reg)}$ is the proportion of diagnoses by provider *k* initiating treatment with regimen *reg* (see below for the construction of this term for different regimens); $\tau^{(reg)}$ is the per-capita rate of treatment completion for regimen *reg* (its inverse reflecting the treatment duration); and $v_{k}^{\left( reg \right)}$ is the per-capita rate of treatment default from regimen *reg*, with provider type *k*. The term $\delta_{j}^{\left( reg \right)}$ is an indicator function, to capture the primary acquisition of drug resistance while on first-line treatment. It takes values:

$\delta_{j}^{\left( reg \right)}=\left\{ \begin{matrix} -1 & \text{if} j=0 \left( \text{DS-TB} \right)\text{ and} reg=FL \\ 1 & \text{if} j=1\left( \text{RR-TB} \right)\text{ and } reg=FL \\ 0 & \text{otherwise} \end{matrix} \right.$

*Bacteriological suppression and relapse:*

Taking treatment completions as a baseline, we assume that those defaulting from treatment, or undergoing spontaneous cure, have an elevated risk of relapse in the first two years following bacteriological suppression. We denote the former state as *R* and the latter as *Q.*

Amongst treatment completions:

$$\dot{R}_{ij}^{\left( reg \right)}=\left[ \sum_{k} c_{j}^{\left( reg \right)}\tau^{\left( reg \right)}T_{ijk}^{\left( reg \right)} \right]-\left( \gamma^{\left( lo, reg \right)}+\omega\lambda_{ij}+s+\mu\right)R_{ij}^{\left( reg \right)},$$

where $c_{j}^{\left( reg \right)}$ is the proportion successfully cured amongst those *completing* treatment with regimen *reg* while having disease with strain *j*; and $\gamma_{ij}^{\left( lo, reg \right)}$ is the per-capita hazard rate of relapse in the first two years of bacteriological suppression, the superscript ‘lo’ designating this as a low relapse risk compartment. This baseline relapse risk depends on the regimen *reg*, as we assume that a future pan-TB regimen may be more ‘forgiving’ than current regimens. Finally, most relapse occurs in the 1-2 years following treatment; we assume that the hazard of relapse ‘stabilises’ to a lower value after this period. The parameter *s* governs this rate of stabilisation.

Amongst treatment defaults:

$$\dot{Q}_{ij}^{\left( reg \right)}= \left[ \sum_{k} c_{j}^{\left( reg \right)}v_{k}^{\left( reg \right)}T_{ijk}^{\left( reg \right)} \right]-\left( \gamma^{\left( hi, reg \right)}+\omega\lambda_{ij}+s+\mu\right)Q_{ij}^{\left( reg \right)},$$

Amongst those who have undergone relapse-free survival, post-treatment:

$$\dot{C}_{ij}=s\left[ \sum_{(reg)} \left( Q_{ij}^{\left( reg \right)}+R_{ij}^{\left( reg \right)} \right) \right]-\left( \gamma^{\left( stabilised \right)}+\omega\lambda_{ij} \right)C_{ij},$$

where $\gamma^{(stabilised)}$ is the per-capita hazard rate of relapse amongst those who have ‘stabilised’ post-treatment.

*Temporarily disengaged from care (failed diagnosis/treatment, or linkage to care)*

$$\dot{E}_{ij}=r_{Dx}\left( 1-\sum_{k,reg} \rho_{ijk}^{\left( reg \right)} \right)D_{ijk}+\left[ \sum_{k,reg} \left( 1-c_{j}^{\left( reg \right)} \right)\tau^{\left( reg \right)}T_{ijk}^{\left( reg \right)} \right]-\left( e+\mu_{TB}+\sigma\right)E_{ij},$$

where *e* is the mean per-capita rate of repeat careseeking.

Force-of infection

For the force-of-infection $\lambda_{ij}$ acting on setting *i* by strain *j*, we have:

$$\lambda_{ij}=\sum_{x} \frac{\beta_{x}g_{ix}\left( I_{xj}+E_{xj}+\sum_{x} D_{xjk} \right)}{N_{x}}$$

where $\beta$ is the number of secondary infections per infectious per year and $N_{x}$ is the total population size in setting *x*. The multiplier $g_{ix}$ takes values: 1 if $i=x$ and *h* if $i\neq x$, for some parameter *h* governing the connectedness between urban and rural settings. We take a plausible but wide range of values *h* (see Table S2).

Treatment initiation proportions

Here we provide further specification of the terms $\rho_{ijk}^{\left( reg \right)}$ in the above equations, or the proportions ultimately initiating first-line, second-line and pan-TB regimen treatment, amongst symptomatics presenting for care. These proportions depend on a combination of factors: drug sensitivity status (DS or RR-TB); the probability of TB diagnosis ($p^{\left( Dx \right)})$; the proportion of cases getting drug sensitivity testing in advance of treatment ($p^{\left( DST \right)}$); initial loss to follow-up (*v*); and the extent of pan-TB regimen deployment. Apart from the first factor, all of these additionally depend on the type of provider involved (*k*).

In particular, amongst symptomatics presenting for care with a provider of type *k*, the proportion initiating first-line treatment is:

$$\rho_{ijk}^{\left( FL \right)}=\left\{ \begin{matrix} p_{k}^{\left( Dx \right)}\left( 1-p_{k}^{\left( UDR \right)} \right)\eta_{k} & \text{DS-TB} (j=0) \\ p_{k}^{\left( Dx \right)}\left( 1-p_{k}^{\left( UDR \right)} \right)\left( 1-p_{k}^{\left( DST \right)} \right)\eta_{k} & \text{RR-TB} (j=1) \end{matrix} \right.$$

Where $p_{k}^{\left( Dx \right)}$ is the probability of correct diagnosis; $p_{k}^{\left( DST \right)}$ is the proportion of treatment initiations being accompanied by a rifampicin sensitivity test to inform treatment; $\eta_{k}$ is the proportion of diagnoses that initiate TB treatment; and $p_{k}^{\left( UDR \right)}$ is the proportion of provider type *k* that use the pan-TB regimen as a universal indication.

Similarly, the proportion initiating second-line treatment is:

$$\rho_{ijk}^{\left( SL \right)}=\left\{ \begin{matrix} 0 & \text{DS-TB} (j=0) \\ p_{k}^{\left( Dx \right)}\left( 1-p_{k}^{\left( UDR \right)} \right)p_{k}^{\left( DST \right)}\left( 1-q_{k}^{\left( UDR \right)} \right)\eta_{k} & \text{RR-TB} (j=1) \end{matrix} \right.$$

where $q_{k}^{\left( UDR \right)}$ is the proportion of provider type *k* that use the pan-TB regimen as an RR-only indication.

The proportion initiating treatment with the pan-TB regimen is:

$$\rho_{ijk}^{\left( UDR \right)}=\left\{ \begin{matrix} p_{k}^{(Dx)}p_{k}^{\left( UDR \right)}\eta_{k} & \text{DS-TB} (j=0) \\ p_{k}^{\left( Dx \right)}\left[ p_{k}^{\left( UDR \right)}+\left( 1-p_{k}^{\left( UDR \right)} \right)p_{k}^{\left( DST \right)}q_{k}^{\left( UDR \right)} \right]\eta_{k} & \text{RR-TB} (j=1) \end{matrix} \right.$$

**Table S1. List of compartments in the model, and their meaning.**

| Compartment | Meaning |
| --- | --- |
| $\boldsymbol{U}_{\boldsymbol{i}}$ | Uninfected in setting *i* |
| $\boldsymbol{L}_{\boldsymbol{ij}}$ | Latent infection in setting *i*, with drug sensitivity *j* |
| $\boldsymbol{I}_{\boldsymbol{ij}}$ | Active disease, before first presentation for care |
| $\boldsymbol{D}_{\boldsymbol{ijk}}$ | Awaiting diagnosis from provider type *k* |
| $\boldsymbol{T}_{\boldsymbol{ijk}}^{\left( \boldsymbol{reg} \right)}$ | Undergoing treatment with regimen (*reg*) with provider type *k* |
| $\boldsymbol{R}_{\boldsymbol{ij}}^{\left( \boldsymbol{reg} \right)}$ | Recovered post-treatment with regimen (*reg*); low relapse risk owing to treatment completion |
| $\boldsymbol{Q}_{\boldsymbol{ij}}^{\left( \boldsymbol{reg} \right)}$ | Recovered post-treatment with regimen (*reg*); high relapse risk owing to treatment default |
| $\boldsymbol{C}_{\boldsymbol{ij}}$ | Stabilised relapse risk following 2 years recurrence-free survival |
| $\boldsymbol{E}_{\boldsymbol{ij}}$ | Temporarily disengaged from care (from failed diagnosis or treatment, or pre-treatment loss to followup) |

**Table S2. List of key parameters in the model.** Footnotes: ^1^ See Figure S2 for sensitivity analysis. ^2^ Public only, as we assume no management of RR-TB, or deployment of pan-TB regimen, in the (unengaged) private sector.

| Parameter | Symbol | | Value | Source/Notes |
| --- | --- | --- | --- | --- |
| Epidemiological data (for model calibration) | | | | |
| Annual risk of TB infection | | Urban | 1.7%  [1.3 - 2.0] | (1, 2) |
|  |  | Rural | 0.9%  [0.7 – 1.1] |  |
| Prevalence of TB disease | | Urban | 203 [163 – 244] per 100k |  |
|  |  | Rural | 338 [271 – 406] per 100k |  |
| Proportion RR-TB amongst incident cases | | | 3.4%  [2.8 – 4.5] | (3) |
| TB natural history | | | | |
| Average infections per case per year | $\beta_{0}$*, urban* | | 20.4  [14.6 – 28.5] | Fitted to ARTI, prevalence data shown above |
|  | $\beta_{1},$ *rural* | | 0.77  [1.61 – 2.69] |  |
| Proportion of new infections being ‘rapid’ progressors to disease | $p_{Fast}$ | | 0.1  [0.08 – 0.12] | (4) |
| Amongst latent infection, per-capita rate of breakdown to active disease | *r* | | 0.001  [$5\times{10}^{-3}-0.002]$ | (5) |
| Per-capita hazard rate, TB mortality | $\mu_{TB}$ | | 0.16  [0.13 – 0.20] | Chosen to yield case fatality rate of 50% (aggregated over smear positive and negative), over an average of three years (6) |
| Per-capita hazard rate, TB spontaneous cure | $\sigma$ | | 0.16  [0.12 – 0.20] |  |
| Reduced susceptibility to infection owing to previous exposure | $\omega$ | | 0.2  [0.1 – 0.3] | Corresponds to 80% reduction in progression as a result of previous infection (7) |
| Healthcare utilisation | | | | |
| Per-capita rate of initial presentation for care | $d_{0}$*,* urban | | 12.9  [8.2 – 19.3] | Fitted to ARTI, prevalence data shown above |
|  | $d_{1},$ rural | | 0.55  [0.23 – 1.02] |  |
| Per-capita rate of repeat presentation for care, following loss from care cascade | *e* | | [10 - 50] | Assumption, subject to uncertainty analysis: corresponds to delay of between 1 week and 1 month (8, 9) |
| Proportion of TB symptomatics visiting the public/private sector for care | $w_{0}$, public | | 0.5  [0.4 – 0.6] | Calibrated to agree with notifications from the public sector |
|  | $w_{1}$, private | | $w=1-w_{0}$ |  |
| TB services | | | | |
| Probability of TB diagnosis per encounter with provider | $p_{0}^{\left( Dx \right)}$*,* public | | 0.84  [0.8 – 0.9] | (10) |
|  | $p_{1}^{\left( Dx \right)}$*,* private | | 0.7  [0.6 – 0.8] | Assumption, subject to sensitivity analysis ^1^ |
| Probability of treatment initiation given diagnosis | $\eta_{0}$*,* public | | 0.87  [0.84 – 0.91] | (10) |
|  | $\eta_{1}$*,* private | | 0.7  [0.6 – 0.8] | Assumption, subject to sensitivity analysis ^1^ |
| Proportion of treatment initiations being guided by DST | $p_{0}^{\left( DST \right)}$*,* public | | 0.35  [0.3 – 0.4] | (11) |
|  | $p_{1}^{\left( DST \right)}$*,* private | | 0 | Assumption |
| Per-capita treatment course rate | $\tau^{\left( FL \right)}$*,* first-line regimen | | 2 | Corresponds to treatment duration of 6 months |
|  | $\tau^{\left( SL \right)}$*,* second-line regimen | | 0.5 | Corresponds to treatment duration of 24 months |
|  | $\tau^{\left( UDR \right)}$*,* pan-TB regimen | | 6 | Corresponds to treatment duration of 2 months |
| Hazard rate of default | $v_{0}^{\left( FL \right)}$*,* public first-line | | 0.13  [0.12 – 0.14] | Corresponds to 6% loss-to-followup (12), allowing for 10% annual variability in hazard rates |
|  | $v_{1}^{\left( FL \right)}$*,* private first-line | | 3.0  [2.4 – 3.6] | With general lack of adherence support in private sector, assuming 40% loss-to-followup, allowing for 20% uncertainty in hazard rates |
|  | $v_{0}^{\left( SL \right)}$*,* public second-line ^2^ | | 0.16  [0.14 – 0.18] | Corresponds to 19% loss-to-followup (12), allowing for 10% annual variability in hazard rates |
|  | $v_{0}^{\left( UDR \right)}$*,* public  pan-TB ^2^ | | 0.13  [0.12 – 0.14] | Assume same hazard rate as for first-line treatment in public sector ($v_{0}^{\left( FL \right)}$) |
| Proportion cure amongst treatment completions | $c_{1}^{\left( FL \right)},$first-line treatment of DS-TB | | 0.98  [0.96 - 1] | 2% failure amongst first-line treatment completions (ref) |
|  | $c_{2}^{\left( FL \right)},$first-line treatment of RR-TB | | 0 | Assumption from inappropriate therapy of RR-TB |
|  | $c_{2}^{\left( FL \right)},$second-line treatment of RR-TB | | 0.77  [0.72 – 0.84] | 13% failure amongst second-line treatment completions (12) |
|  | $c_{1}^{\left( UDR \right)},$pan-TB treatment of DS-TB | | 0.98  [0.96 – 1] | Assumed same as first line treatment of DS-TB ($c_{1}^{\left( FL \right)}$) |
|  | $c_{2}^{\left( UDR \right)},$pan-TB treatment of RR-TB | | 0.98  [0.96 – 1] | Assumed same as outcomes in DS-TB ($c_{1}^{\left( UDR \right)}$) |
| Post-treatment relapse | | | | |
| Relapse hazard rate | $\gamma^{\left( lo,FL \right)}$, following first-line treatment completion | | 0.14  [0.12 – 0.15] | Systematic review of 1-year post treatment outcomes (10) |
|  | $\gamma^{\left( lo,SL \right)}$, following second-line treatment completion | | 0.3  [0.27 – 0.33] |  |
|  | $\gamma^{\left( lo,UDR \right)}$, following pan-TB regimen completion | | 0.07 | Assuming that a pan-TB regimen, by being more ‘forgiving’, can reduce relapse by 50% |
|  | $z$, Relative hazard of relapse, post-treatment default vs completion | | [2 - 3] | (13) Informs post-default relapse rates in relation to post-completion relapse rates listed above as (for example):  $\gamma^{\left( hi,FL \right)}=z{\times\gamma}^{\left( lo,FL \right)}$ |
|  | $\gamma^{\left( stabilisation \right)}$, long-term relapse $\geq$ 2 years post-treatment | | 0.0017 | Chosen to yield 10% lifetime risk of relapse |
| Other model inputs | | | | |
| Per-capita mortality hazard, ‘background mortality’ | $\mu$ | | 1/66 | Chosen to capture a mean lifespan of 66 years (14) |
| Connectivity parameter between urban and rural settings | $h$ | | 0.3  [0.2 – 0.4] | Assumption^1^. See ‘Force-of-infection’, described in supplementary text. |

**Calibration**

Evaluating the posterior density

For a given parameter set $\theta$, (consisting of values for the parameters listed in table S2), we projected the TB epidemic in India until 2017 (see below, ‘projecting TB epidemic from $\theta$’). Taking the model-based projections for the calibration targets above, i.e. prevalence and ARTI in urban and rural settings along with the proportion of incident cases being RR-TB, we calculated the posterior density $\pi(\theta)$ as:

$$\pi\left( \theta\right)=L_{ARTI,urb}\left[ ARTI_{urb}\left( \theta\right) \right]{\times L}_{ARTI,rur}[ARTI_{rur}(\theta)]\times$$

$$L_{Prev,urb}\left[ {Prev}_{urb}\left( \theta\right) \right]\times L_{Prev,rur}\left[ {Prev}_{rur}\left( \theta\right) \right]\times L_{pMDR}\left[ p_{MDR}\left( \theta\right) \right]\times\rho(\theta)$$

where, for example, $L_{ARTI,urb}\left[ ARTI_{urb}\left( \theta\right) \right]$ denotes the likelihood function for urban ARTI (that is, $L_{ARTI,urb}(.)$), acting on the model projection for urban ARTI (that is, $ARTI_{urb}\left( \theta\right)$). To construct these likelihoods we evaluated log-normal distributions capturing the central estimates and 95% uncertainty intervals shown in table S2. Finally, $\rho\left( \theta\right)$ in the expression above indicates the joint prior distribution: we adopted independent uniform distributions for the parameters given in table S2, taking the product over all terms to evaluate $\rho(\theta)$. In practice for a given value of $\theta$ we calculated the log-posterior density, thus taking a sum of the logarithm of each of the terms in the right-hand side of the expression above.

Sampling from the posterior distribution

We used Bayesian melding to sample from the posterior distribution (15). In particular, we used the adaptive MCMC method proposed by Haario et al to create 10^5^ parameter sets $\{\theta_{1},\theta_{2},\ldots,\theta_{{10}^{5}}\}$. We discarded the first thousand parameter sets (‘burn-in’), and then selected every 10^th^ set thereafter (‘thinning’) to remove autocorrelation. Using each element of the resulting parameter set, we created an ensemble of model projections under a given intervention scenario (for example, for the percent cases averted by deployment of a pan-TB regimen at a given coverage – as in table 3). We estimated the point value of these model projections using the 50^th^ percentile of this ensemble, and the 95% credible intervals using the 2.5^th^, and 97.5^th^ percentiles.

Projecting TB epidemic from $\theta$

For a given parameter set $\theta,$ we first simulated the introduction of an infective into a disease-free state in the absence of population growth, RNTCP services or RR-TB. We simulated the system to equilibrium; taking this state as an initial condition from 1980, we increased the birth rate to capture a 1.5% annual increase in the population (14). We introduced RR-TB through a non-zero rate of acquisition while on first-line therapy, assumed to act from 1980 onwards. We further modelled the expansion of RNTCP services, as a linear increase in the parameter $w_{0}$ in the equations above, starting from zero in 1997 and reaching its ultimate value in 2007 (16). Projecting the epidemic forward to 2017, we then recorded model projections for prevalence and ARTI in urban and rural settings, as well as the proportion of incident cases that are RR-TB. We used these projections to calculate the posterior density corresponding to $\theta$, as described above (see ‘Evaluating the posterior density’).

**Interventions**

In modelling the introduction of a pan-TB regimen, we assumed a linear scale-up to replace all public sector treatment under a given indication, from 2022 to 2025. In the ‘RR-only’ indication scenario, we modelled a linear increase in the parameter $q_{k}^{\left( UDR \right)}$ (see equations above) from 0 to 1, over this time period. In the ‘universal indication’ scenario, we instead modelled a linear increase in the parameter $p_{k}^{\left( UDR \right)}$.

When modelling private sector as a ‘background’ intervention, we assumed that all private providers successfully engaged by RNTCP have a quality of care equivalent to that in the public sector. For simplicity we therefore modelled private sector engagement as a linear increase, from 2019 to 2022, in the parameter $w_{0}$ (the proportion of symptomatics visiting the public sector for care) from its initial value $w_{0}(0)$ to a value $w_{0}\left( 0 \right)+0.85[1-w_{0}\left( 0 \right)].$ This choice of parameters represents an intervention that recruits sufficiently many private providers to reach 85% of TB patients currently being managed by the private sector.

**Figure S1.** Model fits to calibration data shown in table S2. Plots show superimposed trajectories over the parameter sets sampled in the Bayesian melding process (grey), compared with the relevant data (black, with errorbars).

**Parameter sensitivity analysis**

We refer to model ‘inputs’ as all quantitative inputs into the model, i.e. both data and parameters (as listed in table S2). As described above, we used Bayesian melding to incorporate uncertainty in the various parameter inputs, and to propagate this uncertainty systematically to model projections. The samples thus obtained from the posterior distribution allow us to perform a multivariate sensitivity analysis, in order to identify the model inputs that are most influential for model outputs. For the purpose of this analysis we selected a specific model output: the threshold cost, under a universal indication, at which a pan-TB regimen is cost-saving to the programme (point *B* in Figure 2). Figure S2 shows results of a partial rank correlation between this and model inputs, the latter ranked from top to bottom in order of decreasing influence. The figure illustrates that the inputs with the most important uncertainties relate to estimates of epidemiological burden: particularly, prevalence and ARTI in urban settings. Amongst those parameters in table S2 that are specified by assumption in the absence of systematic data (principally those parameters relating to the private sector), the most influential is the rate of initial loss-to-followup in the private sector. We note that the model projections shown in the main text simultaneously incorporate the assumed uncertainty in all of these model inputs: however, improved precision in the leading inputs identified by Figure S2 will yield the greatest improvements in these output uncertainty estimates.

**Figure S2. Results of multivariate sensitivity analysis.** Figure shows model inputs, ordered from most influential parameters (top of figure) to least (bottom). ‘ILTFU’ denotes ‘Initial loss-to-followup’; ‘ARTI’ denotes ‘annual risk of TB infection’.

**References**

1. Pandey S, Chadha VK, Laxminarayan R, Arinaminpathy N (2017) Estimating tuberculosis incidence from primary survey data: a mathematical modeling approach. *Int J Tuberc Lung Dis* 21(4):366–374.

2. Central TB Division India Report of the Joint Monitoring Mission. Available at: https://tbcindia.gov.in/index1.php?lang=1&level=1&sublinkid=4710&lid=3264 [Accessed November 3, 2018].

3. World Health Organization Global tuberculosis report 2018. Available at: http://www.who.int/tb/publications/global_report/en/ [Accessed October 29, 2018].

4. Vynnycky E, Fine PE (1997) The natural history of tuberculosis: the implications of age-dependent risks of disease and the role of reinfection. *Epidemiol Infect* 119(2):183–201.

5. Horsburgh CR, et al. (2010) Revisiting rates of reactivation tuberculosis: a population-based approach. *Am J Respir Crit Care Med* 182(3):420–5.

6. Tiemersma EW, van der Werf MJ, Borgdorff MW, Williams BG, Nagelkerke NJD (2011) Natural History of Tuberculosis: Duration and Fatality of Untreated Pulmonary Tuberculosis in HIV Negative Patients: A Systematic Review. *PLoS One* 6(4):e17601.

7. Andrews JR, et al. (2012) Risk of progression to active tuberculosis following reinfection with Mycobacterium tuberculosis. *Clin Infect Dis* 54(6):784–91.

8. Mistry N, Lobo E, Shah S, Rangan S, Dholakia Y (2017) Pulmonary tuberculosis in Patna, India: Durations, delays, and health care seeking behaviour among patients identified through household surveys. *J Epidemiol Glob Health* 7(4):241–248.

9. Mistry N, et al. (2016) Durations and Delays in Care Seeking, Diagnosis and Treatment Initiation in Uncomplicated Pulmonary Tuberculosis Patients in Mumbai, India. *PLoS One* 11(3):e0152287.

10. Subbaraman R, et al. (2016) The Tuberculosis Cascade of Care in India’s Public Sector: A Systematic Review and Meta-analysis. *PLOS Med* 13(10):e1002149.

11. World Health Organization (2017) Global tuberculosis report 2017. Available at: http://www.who.int/tb/publications/global_report/en/ [Accessed September 2, 2018].

12. Revised National Tuberculosis Control Programme RNTCP Annual Status Report 2018. Available at: https://tbcindia.gov.in/showfile.php?lid=3314.

13. Menzies D, et al. (2009) Effect of duration and intermittency of rifampin on tuberculosis treatment outcomes: A systematic review and meta-analysis. *PLoS Med* 6(9):1–18.

14. The World Bank India demographic data. Available at: https://data.worldbank.org/country/india [Accessed November 14, 2018].

15. Alkema L, Raftery AE, Brown T (2008) Bayesian melding for estimating uncertainty in national HIV prevalence estimates. *Sex Transm Infect* 84(Supplement 1):i11–i16.

16. World Health Organization (2010) A brief history of tuberculosis control in India. doi:ISBN 978 92 4 150015 9.
