# Supplementary material for: The potential deployment of a pan-tuberculosis drug regimen in India: A modelling analysis
Source: PLoS One. 2020 Mar 27;15(3):e0230808. doi: 10.1371/journal.pone.0230808 (PMC7100958; doi:10.1371/journal.pone.0230808)
Supplement: Attachment — Submitted filename: Reviewer_responses.docx [file pone.0230808.s002.docx]

We thank the reviewers for their constructive and helpful comments. We have added to the text, as well as including additional analysis, in response these comments. We hope the reviewers will agree, that these changes have helped to improve the manuscript. Point-by-point responses are as follows.

**Reviewer #1:** [Omitting opening comments]

4) The results presented in the main text present a very favorable case for the use of pan-tuberculosis regimens and deployment to all TB cases. In my view, this reflects a rather optimistic view on both the manner in which the new tuberculosis regimen may be adopted (reflected in the treatment initiation parameter) as well as the rates of treatment completion and the actual performance characteristics of the regimen (forgiveness to missed doses, efficacy for RR-TB). I recognize that there is a complex matrix of questions that can be asked, and I don’t expect that the authors would likely choose to address all of these, but several of the following concerns strike me as important to present a more balanced case for both funders and drug developers to consider.

a. While the authors conduct additional analysis in which they sequentially eliminate one of these 4 regimen properties to estimate the impact of a regimen that fails to meet a single one of these properties, it would seem of interest to ask the reverse: What would be the impact of regimens that meet only one of these properties?

*Thank you for this comment – we have incorporated additional analysis as the reviewer suggests. Please see p.9 for a description of this analysis (“…we performed analysis for two types of less-than-ideal regimens…”), and Figure 3 for the updated results.*

b. The assumption of a 2 month regimen seems deserving of a sensitivity analysis – what if the regimen only could be shortened to 4 months for example?

*We have now incorporated additional sensitivity analyses to address this point. While Figure 3 focuses on epidemiological impact, in our new analysis, we extend this analysis to examine the impact on the threshold cost for budget neutrality. In this extended analysis, we also incorporate the key regimen attributes emerging from Figure 3. Please see new text on p.10 (“Concentrating on the threshold regimen costs…”), and new Figure 5 for the corresponding results.*

c. The assumptions around the forgiveness of the regimen to missed doses strike me as a particular example of optimistic thinking and I would like to understand if there is more evidence in the literature to suggest that new regimens are likely to this property (and if so, how dramatic it will be). In my view, this is of critical importance especially since the authors found that this property was the most important for projections of reduction in overall TB burden. The fact that improving outcomes for all cases might be the most important feature of a new regimen does not surprise me and seems in line with other modeling of new regimens (eg Kendall et al PLoS Med 2017), but the idea that a new shorter regimen will be substantially better (2x) in reducing recurrence rate seems dramatic.

*In the absence of systematic evidence from the literature, we have instead opted to explicitly show the sensitivity of model projections to these regimen properties. As described in response to the previous comment, please see Figure 5 for the results of this analysis, as well as p.10 for main text narrating this analysis.*

d. The lack of attention to the potential for emergent resistance to the new regimen is a real limitation. I realize this would seriously compromise the simplicity of the work, but stands out as a feature of concern given that one of the most serious concerns about DST-free regimens is this potential (as described here: Acquah and Furin Lancet Infect Dis 2019 and Dheda et al Lancet Resp Med 2018.). The authors do mention this as a limitation, but I think some additional text linking to some of the dissenting literature might help balance out the case and raise readers attention to the active controversy in this area.

*Thank you for this important point. We have added to the discussion to outline some of these issues, and to include additional citations, including those mentioned by this reviewer. Please see p.15, paragraph beginning, ‘The value of a pan-TB regimen…’, as well as the following paragraph, beginning ‘Nonetheless, for any new regimen…’*

**Reviewer #2:**

This paper is on a potentially important topic, but I am having a hard time getting past the authors' definition of an "ideal regimen". What is described here is almost a fantasy regimen, and I do not understand how they chose the parameters (other than what is in the WHO target product profile--which is a bit of a "dream" document as well). We are nowhere near having a two-month regimen for any forms of TB, and with the exception of the TRUNCATE TB trial, I am not aware of any studies that are even attempting to look at this. As for a "forgiving regimen" that would allow people to miss doses with no implications for their outcomes or for the generation of resistance, this is a wonderful notion, but nowhere on the horizon that I can see. As for assuming that 95% will initiate therapy, there is no evidence that a regimen change alone will lead to increased case detection and initiation of therapy. And the authors also seem to assume this ideal regimen will not be associated with any adverse events and that there will be no pre-existing resistance to the medications in the regimen (which is not true for any drug--there are almost always naturally occurring mutations that are selected for with inadequate therapy). I am not sure such a regimen is even possible, yet these assumptions form the underpinnings of the entire modeling exercise. Why did the author select parameters that are so far outside the realm of what is possible to accomplish in the next decade in TB? If the paper wants to dream the impossible, why not a two week regimen? Some detailed justification would have to be given for why the authors selected the features of the "ideal regimen" that they did--beyond that this is part of a TB target product profile. Otherwise, it could appear that they selected regimen parameters that would give them the results they wanted. I realize there is room for adjusting parameters in modelling work--and in fact changing these parameters can be important in understanding the impact of certain interventions. But what the authors have done here really seems to strain the bounds of what is possible even in the most ambitious and optimistic views of the TB treatment landscape.

*We thank the reviewer for their constructive comments on our analysis, which also raise some important issues in the discussion around pan-TB regimens.*

*We have expanded our analysis to incorporate some additional sensitivity analysis on key model assumptions for regimen characteristics. This now includes analysis of the treatment initiation rate (as highlighted by this reviewer), along with regimen duration and forgiveness (two additional characteristics identified in reviewers’ comments). We show how model findings would change, when less ambitious scenarios are adopted for each of these characteristics. Please see p.10, “Concentrating on the threshold regimen costs…”, and new results shown in Figure 5.*

*On the other points touched on by the reviewer above and elaborated on below, we have added to the text to ensure that these issues are discussed more fully. Please see further details in responses below.*

Second, the authors do not take into account the down sides of dismantling systems for carrying out drug susceptibility testing. By their own admission, this "universal regimen" would likely only work for several years before there was significant resistance. But with the goal of "obviating the need for DST", what would happen then? Would we not be back in the same situation we are when we gave the universal regimen of INH, RIF, PZA, and EMB and did away with DST? What would be the costs of needing to rebuild a system wherein DST could then be done to the drugs in the "ideal regimen". Exploring this may not be possible in the model but should surely be raised in the discussion section.

*An important point, that we now elaborate on more fully, in the discussion. Please see p.15, paragraph beginning “Nonetheless, for any regimen….”.*

Third, the authors leave out any consideration of adverse events and the costs associated with those. In the absence of DST, there will be people who receive medications that their M. tuberculosis strain is resistant to. This is inevitable. And these individuals receiving nothing from those medications but toxicity. Perhaps the "ideal regimen" that the authors want to use also has no associated adverse events? But this is not ever going to be reality. So what about the costs of these unnecessary adverse events? Even if they cannot build them into the model, this issues should surely be mentioned in the discussion section.

*We have now added to the discussion to mention these issues. Please see p.16, paragraph beginning “Another important consideration…”*

Fourth, I am also puzzled as to why they would not account for the selection and amplification of drug resistance. This is a major problem with the use of "universal regimens" and must be accounted for in the model. The development and propagation of rifampicin resistance was a major problem with the use of the universal INH, RIF, PZA, and EMB regimen deployed without DST. Developing a model that pretends this is not relevant or not a risk perpetuates a fallacy and weakens the arguments in favor of a universal regimen.

*We hope the additional text in the discussion now addresses this point more fully. Please see p.15, paragraph beginning “The value of a pan-TB regimen…”*

There are also multiple smaller issues with this paper. First, the use of the term defaulter is stigmatizing and should be avoided.

*We have now updated this language in accordance with Stop TB Partnership recommendations for TB terminology. Please see:*

*“…loss-to-followup…” on ps. 6 and 25, and*

*“….treatment non-completion…” on p.26*

Second, the authors are citing literature incorrectly. For example, they cite the Schnippel et al paper from South Africa that showed a decreased mortality when bedaquiline was given to people living with RR-TB. Yet they claim this paper shows a "reduced mortality with shorter regimens." If anything, the data published on shorter regimens (most notably the Nunn et al trial published in the New England Journal of Medicine) showed a trend toward higher mortality among people treated with a shorter regimen, especially if they were also living with HIV.

*We have adjusted this narrative to remove reference to mortality rates associated with shorter regimens. Please see p.3, “…new, shortened regimens for multi-drug-resistant TB…”*

I think a modeling paper on this topic could be very important and interesting. But it has to have some basis in reality and it cannot exclude "inconvenient" parameters that would most certainly play a role in the effectiveness of such a regimen (including side effects and resistance selection).

*We thank the reviewer for their comments, and hope they will agree that our responses have helped to improve the paper.*
