# Supplementary material for: The potential deployment of a pan-tuberculosis drug regimen in India: A modelling analysis
Source: PLoS One. 2020 Mar 27;15(3):e0230808. doi: 10.1371/journal.pone.0230808 (PMC7100958; doi:10.1371/journal.pone.0230808)
Supplement: Attachment — Submitted filename: Reviewer_responses.docx [file pone.0230808.s003.docx]

**ACADEMIC EDITOR:**

I see that the abstract has not been revised to reflect the more balanced view of the paper. Please add some text around the assumptions and limitations of the study.

*We have now added to the abstract to elaborate on the model limitations, focusing on the important discussion around mitigating drug resistance, and the need for DST. Please see p.2, “One limitation of our approach…”*

There is a typo in line 289 'forimpact'.

*Thank you for spotting this – now corrected.*

Lines 328-329: I think it might be good to temper the statement the a pan-TB regimen will likely to be useful for several years. There is limited evidence that the introduction of fixed dose combinations and adherence technologies have reduced the risk of resistance acquisition.

*We have reworded accordingly – please see p.15, “To the extent that…”*

**Reviewer #2:**

Thank you for updating your paper to address all the comments and concerns. It is a stronger paper now and I think more based in reality. I have only one small comment. In the paper there appears to be a missing reference--I think the authors meant to put one in here but it just says "ref".

*We are glad the reviewer feels the paper has been improved, and appreciate their constructive comments. Thank you for spotting the reference placeholder, which is now fixed. Please see p.15.*

While I agree with the overall message here, the authors should still note that one important strategy that has been used to slow the emergence of drug resistance has drug-susceptibility testing at the time of TB diagnosis. This is effective and is standard of care in wealthy countries. Non-adherence is not the only cause of emerging drug resistance but so is inadequate therapy. The authors need to acknowledge this.

*Please note that we discuss the need for DST in the very next paragraph. Nonetheless we have now reworded this discussion, to emphasise the value of DST-guided therapy in slowing the emergence of drug resistance, and to link this with the discussion in the preceding paragraph. Please see p.15, “With current anti-TB regimens…”*
